# Author’s reply

**DOI:** 10.4103/1817-1737.74280

**Published:** 2011

**Authors:** R. Garg

**Affiliations:** *Department of Pulmonary Medicine, Chhatrapati Sahuji Maharaj Medical University, Lucknow-226003. Uttar Pradesh, India. E-mail: rajivkgmc@gmail.com*

Sir,

This is in reference to the letter by an esteemed reader. I appreciate the interest shown by him regarding our case. We regret that the case reported by him was wrongly quoted in our article as caused by *Mansonella perstans* and apologize for the inadvertent error. The only reported case of pleural effusion due to *M. perstans* was of Kahn in 1983.[1]

We did not consider pleural biopsy as the diagnosis was firmly established by repeated demonstration of microfilariae in pleural fluid, and the patient denied consent for pleural biopsy. The literature shows that pleural biopsy does not always demonstrate microfilariae.[2,3]

The method used for demonstration of microfilariae in pleural fluid was hematoxylin and eosin stain on smears prepared from the sediment of pleural fluid after centrifuging at 2000 rpm for 15 min. Pleural fluid samples were collected at night time or in early morning.
